# Developing a questionnaire to evaluate the health information literacy in China

**DOI:** 10.3389/fpubh.2023.1068648

**Published:** 2023-06-16

**Authors:** Xuan Yu, Meng Luo, Shouyuan Wu, Juanjuan Zhang, Qiangqiang Guo, Xiaohui Wang, Youzhong Tian, Zhizhong Zhang, Yaolong Chen, Jianqiang Wen

**Affiliations:** ^1^Evidence-Based Medicine Center, School of Basic Medical Sciences, Lanzhou University, Lanzhou, Gansu, China; ^2^School of Public Health, Lanzhou University, Lanzhou, Gansu, China; ^3^Center for Disease Control and Prevention, Jiuquan, Gansu, China; ^4^Health Propaganda and Education Center of Gansu, Lanzhou, China; ^5^Research Unit of Evidence-Based Evaluation and Guidelines, Chinese Academy of Medical Sciences (2021RU017), School of Basic Medical Sciences, Lanzhou University, Lanzhou, Gansu, China; ^6^WHO Collaborating Centre for Guideline Implementation and Knowledge Translation, Lanzhou, Gansu, China; ^7^Gansu Provincial Centre for Development of Traditional Chinese Medicine, Lanzhou, China

**Keywords:** health information literacy, questionnaire development, reliability and validity, COVID-19 pandemic, evidence-based

## Abstract

**Introduction:**

Health information literacy is critical for individuals to obtain, understand, screen, and apply health information. However, there is currently no specific tool available to evaluate all four dimensions of health information literacy in China. Public health emergencies can present an opportunity to evaluate and monitor the health information literacy level of residents. Therefore, this study aimed to develop a questionnaire to evaluate the level of health information literacy and to measure the reliability and validity.

**Methods:**

The development process of the questionnaire consisted of the determination of questionnaire items, expert consultation, and validation. Based on the National Residents Health Literacy Monitoring Questionnaire (2020) and the 2019 Informed Health Choices key concepts, the researchers drafted the questionnaire, including all four dimensions of health information literacy. Experts in relevant fields were invited to evaluate the draft questionnaire, and revisions were made accordingly. Finally, the reliability and validity of the finalized version were examined in Gansu Province, China.

**Results:**

The research team preliminarily formulated 14 items encompassing the four dimensions of health information literacy. After consulting with 28 experts, modifications were made. A convenience sample of 185 Chinese residents was invited to participate. Cronbach's alpha coefficient was 0.715 and McDonald's omega was 0.739 for internal consistency, and the test-retest intra-class correlation coefficient after 4 weeks was 0.906, indicating that the questionnaire content and measurement structure was relatively stable.

**Conclusion:**

This questionnaire is the first evidence-based assessment tool developed for monitoring health information literacy in China, and it has shown good reliability and validity. It can help to monitor the health information literacy levels of Chinese residents, promote evidence-based decision-making, and guide interventions to improve health information literacy.

## 1. Introduction

Health literacy is defined as the capacity of individuals to obtain, process, and understand basic health information and services when making appropriate health decisions (1, 2). As a branch of health literacy, health information literacy emphasizes a range of information abilities, including recognizing health information needs, identifying possible sources of information, using them to retrieve relevant information, assessing the quality of information and the application of information in a specific situation, and analyzing, understanding, and using information to make scientific health decisions (3). Unlike health literacy, health information literacy combines both health literacy and information literacy, and it could place more emphasis on the human ability to discover and use health-related information (4). Good health information literacy not only can help people acquire more knowledge related to disease prevention and treatment, maintain a healthy lifestyle, improve doctor-patient communication and smooth doctor-patient conflicts, but it can also reduce the waste of health resources, improve personal health, and promote economic growth and social progress while meeting evidence-based practice (5–9).

As education is one of the hot topics in some developed countries during COVID-19 (10), it is crucial to conduct evaluations that provide evidence-based interventions for future health information literacy education. Several international health information literacy evaluation projects have been conducted or are being conducted, such as the Health Information Literacy Research Project by the US National Library of Medicine (3) and the Daily Health Information Literacy Evaluation Projects for Individuals aged 65 and older in Finland (11). However, in China, national research on health information literacy started relatively late. In 2012, the health information literacy of Chinese residents was assessed for the first time in the monitoring of health literacy among Chinese residents, and was investigated and analyzed separately from the dimension of health information literacy for the first time (12). Nie et al. (13) investigated the health information literacy levels of urban and rural residents in six Chinese provinces based on six items from the Health Literacy Questionnaire and found that these residents had poor health information literacy. However, since this survey instrument does not specifically investigate the health information literacy level of residents, the results cannot comprehensively present the level of health information literacy for Chinese residents.

During COVID-19, several investigations of health literacy were conducted in China; however, only one investigation of health information literacy was conducted on patients who tested positive for COVID-19 (14). This limited population study does not provide a clear understanding of the overall health information literacy level of Chinese residents. While various approaches to evaluating health information literacy exist worldwide, they typically focus on one or two aspects of health information literacy and are not comprehensive (15).

Currently, there are no specific tools to evaluate the four dimensions of health information literacy in China, and most evaluations used individual items extracted from existing questionnaires. Evaluation is a prerequisite for researching and improving health information literacy levels, as it helps us quickly understand the current situation of health information literacy among Chinese residents. In addition to the health-related impact, COVID-19 has had far-reaching effects on China's economy (16, 17), environment (18), and technology (19, 20), and it has led to the circulation of several pieces of misinformation affecting the public's judgement (21). Therefore, the COVID-19 pandemic presents an ideal time to evaluate and monitor the level of health information literacy, as it has affected everyone's lives. This study aims to develop an evidence-based tool for evaluating the level of health information literacy among Chinese residents and to measure the reliability and validity of the questionnaire. The purpose of this new questionnaire is to provide a scientific basis for evaluating and monitoring the level of health information literacy among Chinese residents, and to use this information to help them make optimal health choices in the future.

## 2. Methods

### 2.1. Establishment of a questionnaire development working group

The working group for this study comprised decision-makers, researchers, and students from various institutions, including the Health Commission of Gansu Province, the School of Basic Medicine Sciences and the School of Public Health of Lanzhou University, the Gansu Provincial Centre for Development of Traditional Chinese Medicine, and the WHO Collaborating Center for Guideline Implementation and Knowledge Translation. In addition, experts from multiple disciplines such as epidemiology, evidence-based medicine, evidence-based social sciences, public health management, health policy, and health education were recruited to ensure a wide range of perspectives were represented during the development of the questionnaire.

### 2.2. Development process

The development process of the questionnaire consists of three stages: determining the content of the questionnaire items, expert consultation, and validation.

#### 2.2.1. Preliminary questionnaire items

The National Residents Health Literacy Monitoring Questionnaire (NRHLMQ 2020) and the 2019 Informed Health Choices Key Concepts (2019 IHC KCs) were used as the basis for drafting the pool questionnaire items for the new health information literacy questionnaire in this study. The NRHLMQ 2020 aimed to assess the level of health knowledge and skills of Chinese residents, while the 2019 IHC KCs were developed by IHC (IHC aims to help people to think critically when making informed choices related to health) to help people make evidence-informed health choices based on clear and practical criteria. 2019 IHC KCs through a systematic, transparent, and iterative process involving potential end users and relevant experts, while considering treatment effectiveness and evidence (22, 23). The IHC KCs was found to be unique among the 22 frameworks examined in a systematic review by Oxman et al. (24), which critical thinking and evidence-based practice as its core.

The items in the NRHLMQ 2020 were the five questions selected by Nie et al. (13) From the 2019 IHC KCs, five researchers (Yu X, Luo M, Wu SY, Zhang JJ, Guo QQ) selected 15 KCs that fit the four dimensions of health information literacy: initiative in obtaining health information; assessing health information; comprehending and applying health information; and screening health information screening. The five reviewers independently working in pairs (Group 1: Yu X & Luo M; Group 2: Yu X & Wu SY; Group 3: Zhang JJ & Guo QQ) selected IHC KCs based on these four dimensions. Disagreements were resolved through discussion or with the help of a third researcher (Chen YL) if consensus could not be achieved. The working group complied with the preliminary items of the new *Health Information Literacy of Chinese Residents During the COVID-19 Questionnaire* via a consensus process among all members.

#### 2.2.2. Expert consultation

In order to further refine the questionnaire, experts from relevant fields were invited to evaluate the first draft in two rounds of consultation. During the first round, experts were asked to rate the necessity, importance, feasibility, and clarity of each item in the questionnaire using a five-point Likert scale (1 = Strongly disagree, 2 = Disagree, 3 = Undecided, 4 = Agree, and 5 = Strongly Agree). They were also given the opportunity to provide suggestions for modifying specific items or for the overall questionnaire. Based on the results of this round, items with mean scores of ≥3·5 and full score ratios of ≥0·2 were retained, while those with lower scores were either modified or deleted.

During the second round, experts were asked to evaluate the relevance of dimensions to topics and items using a four-level Likert scale (scores of 1 to 4 indicate irrelevant to very relevant). The item-level content validity index (I-CVI) was calculated and corrected for expert probabilistic consistency (Pc), with modified Kappa statistics (K^*^) used to assess the validity of the questionnaire. An I-CVI score of ≥0·78 and a K^*^ value between 0·60 and 0·74 were considered indicators of good content validity, while a K^*^ value of >0·74 indicated excellent validity (25).

#### 2.2.3. Validation

##### 2.2.3.1. Study design

The validation study was conducted in Gansu Province, China, from May 26 to June 26, 2021. To ensure a diverse sample, three cities were selected based on factors such as economic development, geographical location, and population growth. Convenience sampling was used to recruit participants, with consideration given to age, gender, urban-rural ratio, and other characteristics. Initially, a target sample size of 140 participants was identified, which was approximately ten times the number of questionnaire items. However, to account for potential missing interviews and invalid surveys, the sample size was increased by 20%, resulting in a target sample size of more than 168 participants.

The inclusion criteria for respondents were: (1) age 15–69 years of age; (2) Chinese nationals; (3) permanent residents who had lived in Gansu Province for 6 months or longer; (4) normal mental status, no serious hearing or vision problems, and ability to communicate in Chinese; and (5) agreement to participate in this study.

##### 2.2.3.2. Survey instruments and quality control

Eight investigators were recruited and trained in groups of two. The purpose and content of the survey were explained to the respondents. After obtaining their informed consent, the investigators read out the questions one by one, and the respondents answered independently or verbally informed the investigators, who then filled in the responses on their behalf. All questionnaires were collected immediately after completion, and the results were entered into the research database using MS Office Excel on the same day. Yu X verified the questionnaires on the same day they were collected to eliminate irrational responses and incomplete questionnaires and ensure the validity of the data.

### 2.3. Statistical analysis

The degree of expert coordination was evaluated by calculating Kendall's coordination coefficients (W). A significance level of *P* < 0·01 was considered well-coordinated for the criteria of necessity, importance, feasibility, clarity, and the total Kendall coordination coefficient, indicating that the opinions of the experts were well-coordinated and the results were credible. The results of the expert consultation were analyzed using the expert authority coefficient and the degree of coordination. The expert authority coefficient (Cr) is the mean of the judgment basis coefficient (Ca) and the familiarity coefficient (Cs). The data in the validation study were analyzed using SPSS 26.0. Frequency and percentage were used to describe counting data, while mean ± SD was used for quantitative data. Content validity, internal consistency, and test-retest reliability were evaluated using the item-level content validity index (I-CVI), Cronbach's alpha coefficient, McDonald's omega, and intraclass correlation coefficient (ICC), respectively. Structural validity was assessed using exploratory factor analysis (EFA). The answers of the survey participants for all items have been converted into binary variables (correct answers were recorded as “1” and incorrect answers were recorded as “0”). Meanwhile, the outcome variable was divided into “adequate health literacy” and “inadequate health literacy” using a cutoff of 70% of the total score.

### 2.4. Ethics approval

The study was conducted in full compliance with the principles of voluntariness, confidentiality, and respect for human subjects, protecting the legitimate rights and interests of the respondents. This study was approved by the Ethics Committee of the School of Public Health, Lanzhou University, China (Approval Number: IRB21032901).

## 3. Results

### 3.1. Questionnaire development

**Figure 1:**
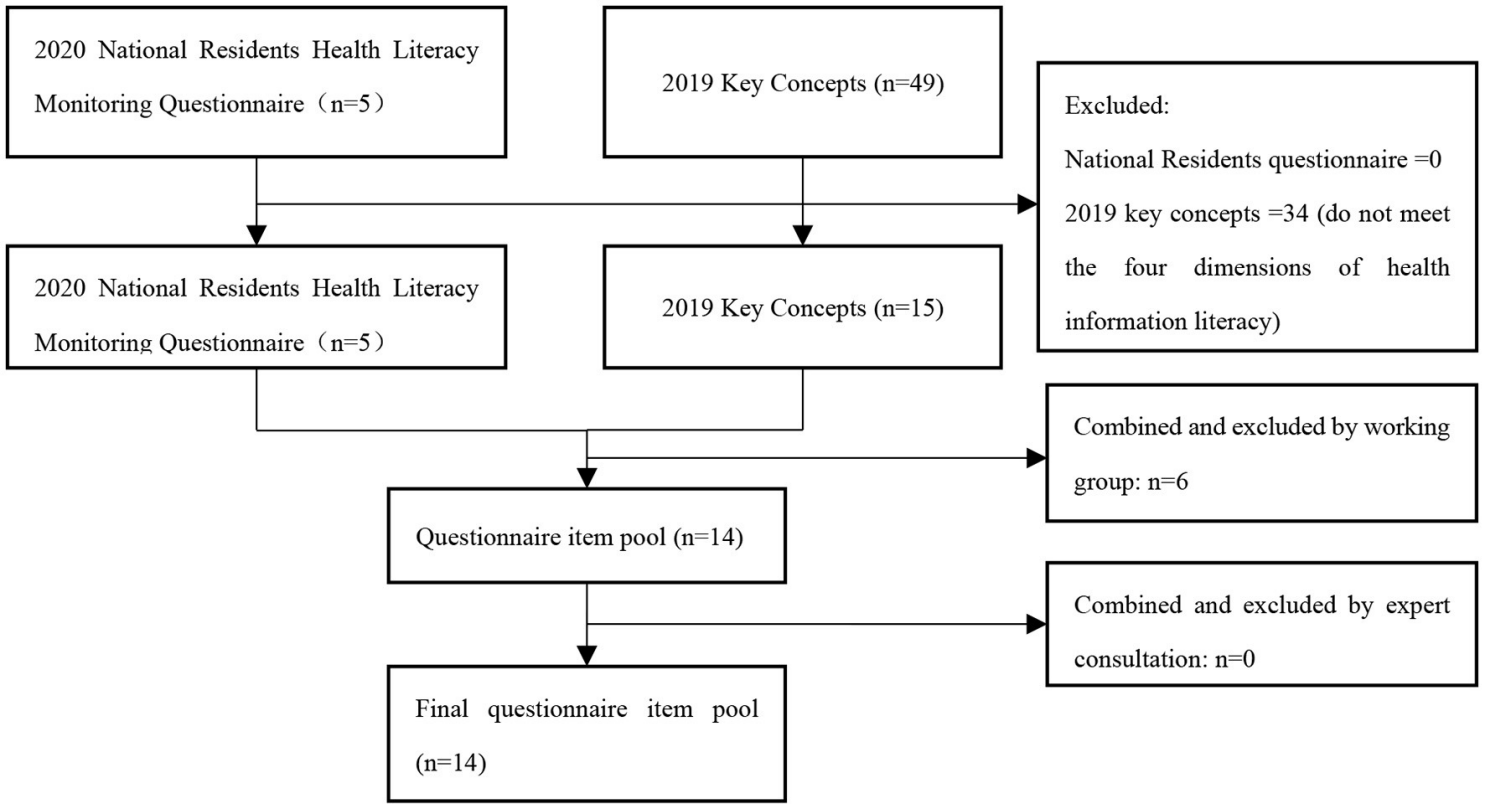
Item pool development process.

The research team developed a questionnaire consisting of 14 items to assess health information literacy across all four dimensions. These dimensions included health information initiative (one item adapted from the NHLMQ 2020), evaluating health information (one item adapted from the 2019 IHC KCs), health information comprehension and application ability (ten items adapted from the 2019 IHC KCs), and health information screening ability [two items adapted from the Technical Guideline to COVID-19 Vaccination (First Edition)]. All questions were presented as single-choice questions. The questionnaire item development process is described in detail in Figure 1, and the final version of the questionnaire is included in Appendix 1.

### 3.2. Expert consultation and content validity

A total of 28 experts in related fields were consulted in the first round, including experts in national health education, health communication, behavioral science, public health policy, health promotion, public policy, disease prevention and control, economics, health career management, epidemiology, basic medical education research, and nutrition and health. The characteristics of these experts are shown in Table 1.

**Table 1:** Characteristics of the experts in the first round.

**Characteristic**	**No. of Experts**	**Proportion (%)**
**Gender**		
Male	17	60.7
Female	11	39.3
**Age (years)**		
26~30	1	3.6
31~40	5	17.9
41~50	9	32.1
51~60	13	46.4
**Education**		
Bachelor's degree and below	7	25.0
Master's degree	10	35.7
Doctorate degree	11	39.3
**Professional title**		
Senior	16	57.1
Vice-senior	5	17.9
Middle	3	10.7
Primary	1	3.6
Others	3	10.7
**Workplace**		
Gansu Province, China	17	60.7
Outside of Gansu Province, China	11	39.3

The results of expert self-identification indicated that all 28 experts had high Cr value (0·92), with Ca value ranging from 0·7 to 1·0, and Cs value ranging from 0·8 to 1·0. The degree of expert authority was therefore considered high. The Kendall concordance coefficients (W) values were obtained for each factor, with W _necessity_= 0·088, W _importance_= 0·115, W _feasibility_= 0·168, W _clarity_= 0·172, and W _overall_= 0·139, and the results were statistically significant (*P* < 0·01) indicating consistency among the experts' opinions. However, the consistency was considered moderate to low. Despite this, all items were considered for retention as the mean values across the four dimensions and the full score ratio met the necessary criteria (mean scores of ≥3·5 and full score ratios of ≥0·2). Based on the expert opinions, modifications were made to the questionnaire such as adding a “Don't know” option to each item and revising the options of five items to better suit the questionnaire's purpose and make the questions easier for respondents to understand.

In the second round of expert consultation, 10 experts in health policy, evidence-based medicine, health education, and other related fields were consulted to further evaluate the content validity of the questionnaire. The results showed that the I-CVI of 12 items was >0·78, indicating excellent content validity. The k^*^ value of these items was >0·74, indicating substantial agreement among the experts. Item 4 and item 8 were approved by 7 out of 10 experts, with an I-CVI of 0·70 and a K^*^ of 0·66 after random consistency correction, indicating good content validity. Overall, all items were considered to have at least good content validity and were retained in the final version of the questionnaire.

### 3.3. Validation of the questionnaire

#### 3.3.1. Demographic characteristics of survey respondents

Out of the 204 questionnaires distributed in the field survey, 198 were returned. Of these, 185 were considered valid, resulting in a response rate of 93.43%. The respondents had an average age of 42.73 ± 13.36 years, with a range of 15 to 69 years. The gender distribution was equal. Similarly, the respondents were equally distributed between urban and rural areas, with 50.8% residing in urban areas and 49.2% in rural areas. For further details, please refer to Table 2.

**Table 2:** Demographic characteristics of respondents in the validation study.

**Descriptions**	**No. of respondents**	**Proportions (%)**
**Gender**		
Male	98	53.0
Female	87	47.0
**Household registration**		
Urban	94	50.8
Rural	91	49.2
**City**		
Lanzhou	57	30.8
Dingxi	52	28.1
Jiuquan	76	41.1
**Age**		
15~19	2	1.1
20~29	35	18.9
30~39	45	24.3
40~49	37	20.0
50~59	45	24.3
60~69	21	11.4
**Ethnic**		
Han	179	96.8
Minorities	6	3.2
**Education**		
Illiterate or barely literate	5	2.7
Primary school	31	16.8
Secondary school	37	20.0
High school/professional high school/special secondary school	21	11.4
College	32	17.3
Bachelor's degree	45	24.3
Master's degree	11	5.9
Doctorate degree	3	1.6
**Career**		
Professionals (e.g., teachers/doctors/lawyers, etc.)	19	10.3
Service workers (e.g., caterers/drivers/salesmen, etc.)	10	5.4
Freelancers (e.g., writers/artists/photographers/tour guides, etc.)	2	1.1
Workers (e.g., factory workers/construction workers/urban sanitation workers, etc.)	20	10.8
Company employee	12	6.5
Government institution employee/civil servants/government staff	36	19.4
Student	10	5.4
Housewife	10	5.4
Others (famers)	66	35.7
**Have you been involved in medical science research?**		
**(e.g., medical projects, trials, etc.)**		
Yes	14	7.6
No	171	92.4
**Have you received health science education?**		
**(e.g., health lectures, surveys, etc.)**		
Yes	129	69.7
No	56	30.3
**Are you paying continuous attention to the relevant**		
**information of the COVID-19 pandemic?**		
Yes	176	95.1
No	9	4.9

#### 3.3.2. Accuracy rate analysis

The accuracy rate of the questionnaires distributed in urban and rural areas was analyzed separately. If the accuracy rate of a particular item was found to be below 60%, the options with the highest percentage of wrong choices were examined. The questionnaire development working group then discussed whether revisions to the questionnaire items and options were necessary. If the item itself was found to be unambiguous, the original questionnaire item was retained.

The accuracy rates for items 6, 9, and 12 were below 60% for both urban and rural areas in the 185 questionnaires analyzed. After discussions and consensus among the working group, item 12 was modified, while item 6 and item 9 were retained with linguistic adjustments. In the 94 questionnaires from urban areas, item 4 had an accuracy rate of 58.51%, and the option “C” was chosen incorrectly by 53.20% of respondents. Therefore, a semantic modification was made to option “C.” In the 91 questionnaires from rural areas, item 3, 5, 10, 11, and 13 had error rates higher than 60%. The working group concluded that the high error rates were due to a lack of relevant knowledge and not the ambiguity of the items. Therefore, the original item 3, 10, 11, and 13 were retained, and additional clarification was provided for item 5.

#### 3.3.3. Reliability and validity analysis

The results of the consultation on content validity showed that the proportion of experts who rated the necessity, importance, and feasibility of each item as 3 and above (i.e., the items were considered necessary, important, and feasible) ranged from 82.1 to 96.4%, which represents good content validity (content validity index (CVI) > 0.78).

Structural validity was analyzed using exploratory factor analysis. The KMO value was 0.759 (*P* < 0.01), indicating that the data were suitable for factor analysis. Principal component analysis with maximum variance orthogonal rotation was used to extract four common factors with eigenvalues >1, as indicated by the scree plots. The factor loading scores were >0.4, and the cumulative contribution of the variance of the four factors was 51.47%.

**Figure 2:**
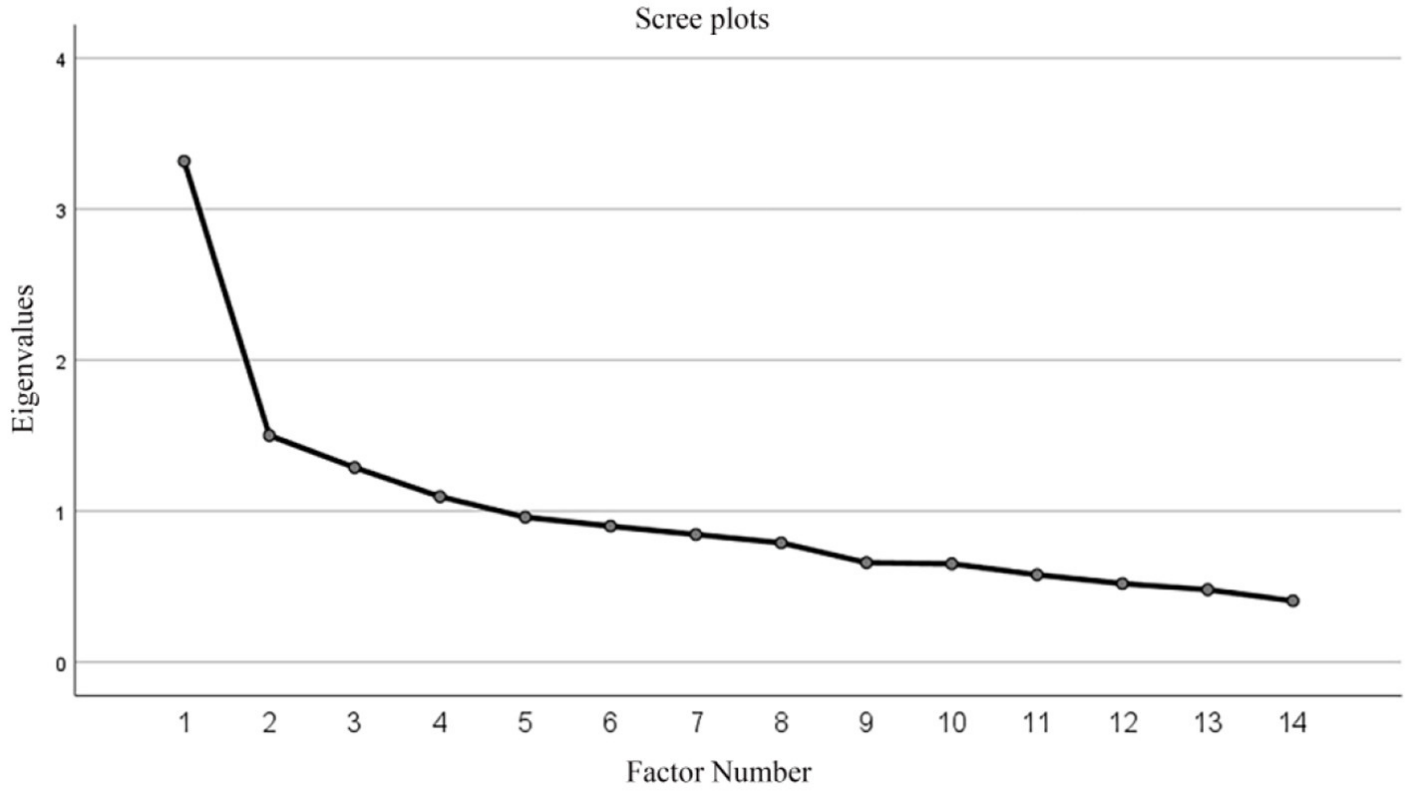
Scree plot.

Factor 1 included item 1, 2, 5, 7, and 10, and it related to the domain of obtaining health information. Factor 2 included item 3, 9, 12, and 14, and it related to the domain of evaluating health information. Factor 3 included item 4 and 8, and it related to the domain of comprehending and applying health information. Factor 4 included item 6, 11, and 13, and it related to the domain of screening health information. The factor analysis results are presented in Figure 2 and Table 3.

**Table 3:** Factor loadings after rotation.

**Item**	**Factor**			
	**1**	**2**	**3**	**4**
Item 7	0.749			
Item 1	0.709			
Item 2	0.648			
Item 10	0.567			
Item 5	0.452			
Item 14		0.680		
Item 9		0.572		
Item 3		0.532		
Item 12		0.466		
Item 8			0.745	
Item 4			0.682	
Item 6				0.782
Item 11				0.496
Item 13				0.427
Eigenvalues	3.318	1.502	1.289	1.097
Variance contribution rate (%)	17.726	13.004	10.407	10.333
Cumulative variance contribution rate (%)	17.726	30.730	41.138	51.470

The results of the reliability analysis showed that Cronbach's alpha coefficient for internal consistency was 0.715 and McDonald's omega was 0.739. To assess the stability of the questionnaire over time, a random sample of 18 participants from the 185 valid questionnaires was selected for retesting 4 weeks after the initial survey, representing 10% of the sample. The answers to all items by the participants were converted to binary variables, with correct answers scored as “1” and incorrect answers scored as “0.” The ICC was analyzed using a two-way mixed model with absolute agreement as the evaluation type. The test-retest ICC value was 0.906 (95% CI, 0.752 to 0.965), indicating high reliability of the retest and good stability of the content and measurement structure of the questionnaire.

## 4. Discussion

As the COVID-19 pandemic continues to place increasing demands on the healthcare system, it has become essential for the public to have the ability to evaluate and apply health information. Consequently, health information literacy has emerged as an important area of research. This study is the first to integrate the evidence-based IHC KCs and the Chinese National Health Literacy Monitoring Questionnaire (2020) to develop a tool with strong reliability and validity. It is suitable for monitoring the health information literacy of Chinese residents during the COVID-19 pandemic in Gansu, China. The questionnaire comprehensively evaluates all four dimensions of health information literacy and each item is supported by reliable evidence.

Although high-income countries such as the United States and the United Kingdom implemented health information literacy education programs at the beginning of the 21st century (26), in China, it has only received gradual attention from relevant scholars since 2012, mainly focusing on health information literacy factors and implementation approaches (27). The currently available surveys on health information literacy of Chinese residents are not comprehensive and mostly focus on specific populations, such as university students and the older adult(s), or limited geographic regions (28, 29). Some studies have analyzed the level of health information literacy of Chinese residents using health literacy monitoring data (13, 30, 31). However, these studies do not fully reflect the health information literacy level of the Chinese residents, indicating the need for more effective tools for monitoring.

Monitoring health information literacy is essential to promote evidence-based practice (9). The *Health Information Literacy of Chinese Residents During the COVID-19 Questionnaire* developed in this study suggests that the level of health information literacy is relatively low in both urban and rural areas. Residents tend to rely more on the recommendations of authoritative experts without deeply considering whether the recommendations were supported by high-quality evidence. In the comparison between a new method or technology and an old one, most residents thought that the new method or technology must be better. During the COVID-19 pandemic, the public also believed that taking more of a drug to be beneficial for prevention would further increase its preventive effect.

It is important to recognize that the findings of this study are based on small sample size and may not be generalizable to the entire population. Therefore, larger and more diverse samples of Chinese residents are needed to validate these results. In the next phase of the study, the working group plans to monitor the level of health information literacy for more than 3,000 residents in Gansu province using this newly developed questionnaire. Based on the results, the working group aims to develop tailored evidence-based health information items for Chinese residents not only in Gansu, but also for other provinces, which can empower the public to make more informed choices regarding their health.

There are some limitations to this study. First, Due to the initial extraction of four dimensions of health literacy using IHC KCs, there is a factor with only two items, which may somewhat diminish the explanatory power of the factor structure and the reliability of the conclusions. Second, the reliability and validity of the questionnaire were based on the population of Gansu Province, and therefore, the applicability of the results to the entire Chinese population cannot be guaranteed. However, our next plan is to focus on the residents of Gansu province to further investigate their level of health information literacy. Second, the sample size of 185 participants may be considered small; however, this study aimed to develop and validate the Questionnaire, and the reliability and validity analyses were conducted appropriately. Third, the study only focused on the level of health information literacy during the COVID-19 pandemic, and did not investigate changes in health information literacy over time or in response to other health crises. Nonetheless, given the unique context of the COVID-19 pandemic, using this questionnaire to investigate public health information literacy is currently the best approach. The working group plans to periodically update the questionnaire, approximately every 3–5 years, to enhance its scientifically rigorous and provide a comprehensive assessment of the public's health information literacy in China.

## 5. Conclusion

The *Health Information Literacy of Chinese Residents During the COVID-19 Questionnaire* is the first assessment tool specifically developed for health information literacy monitoring in China. This study has demonstrated that the questionnaire shows good reliability and validity, making it a valuable tool for monitoring the level of health information literacy for residents. This study contributes to the advancement of health information literacy research and provides insights for policymakers and health professionals to develop more effective strategies to improve health information literacy levels among the public, particularly during public health emergencies like COVID-19.

## Data availability statement

The original contributions presented in the study are included in the article/Supplementary material, further inquiries can be directed to the corresponding authors.

## Ethics statement

The study was conducted in full compliance with the principles of voluntariness and confidentiality, and respect for human subjects and protects the legitimate rights and interests of the respondents. This study was approved by the Ethics Committee of the School of Public Health, Lanzhou University, China (Approval Number: IRB21032901) and has been conducted. The patients/participants provided their written informed consent to participate in this study.

## Author contributions

JW and YC contributed to the conception and design of the study. XY and ML implemented the analysis of data and wrote the first draft of the manuscript. XY, ML, SW, JZ, QG, XW, JW, and YC developed the first draft of the questionnaire. XY, ML, SW, JZ, QG, XW, YT, ZZ, and JW conducted field surveys and entered survey data. All authors contributed feedback on the study results and revised manuscript, contributed to the article, and approved the submitted version.
