# Supplementary material for: Developing a questionnaire to evaluate the health information literacy in China
Source: Front Public Health. 2023 Jun 16;11:1068648. doi: 10.3389/fpubh.2023.1068648 (PMC10311903; doi:10.3389/fpubh.2023.1068648)
Supplement: Supplementary file 1 [file Data_Sheet_1.docx]

**Appendix 1. Questionnaire**

**2021年甘肃省居民新型冠状病毒肺炎健康信息素养问卷**

**尊敬的受访者：**

**您好！我们是甘肃省卫生健康委（原甘肃省卫生厅）的工作人员，现在正在进行甘肃省居民新型冠状病毒肺炎健康信息素养调查，这项调查是省卫生健康委的科研项目。您所填写信息将是本研究的重要数据来源，在您参与调查前，请仔细阅读以下内容。**

**您所填写的所有数据仅供研究使用，绝不外传，请您安心填写。您的回答没有对错之分，只需根据您的实际理解情况填写即可。此外，问卷的所有内容需要您个人独立填写。**

**本研究已获得兰州大学公共卫生学院伦理审查委员会的同意。您的参与对我们的研究非常重要，非常感谢您的协助和参与。如果您有任何关于课题方面的疑问，欢迎及时与我们联系。（联系人：文建强，联系电话：09318649683）**

**甘肃省卫生健康委《新冠肺炎疫情防控背景下甘肃省健康信息素养项目》项目组**

**2021年7月**

1. 基本信息
2. 出生年月： 年 月
3. 性别：A 男 B 女
4. 您的民族：

A汉族 B 回族 C 藏族 D 蒙古族 E 哈萨克族 F 其他（请填写）

1. 您的文化程度：

A 不识字或识字很少 B 小学 C 初中 D 高中/职高/中专 E 大专 F 本科 G 硕士 H博士

1. 您的职业是：

A 专业人士（如教师/医生/律师等） B服务业人员（餐饮服务员/司机/售货员等） C自由职业者（如作家/艺术家/摄影师/导游等） D工人（如工厂工人/建筑工人/城市环卫工人等） E公司职员 F事业单位/公务员/政府工作人员 G学生 H家庭主妇 I其他（商人）

1. 您的户口类型是？

A 城市 B 农村

1. 您目前的所在居住地： 市 区/县/镇

A 兰州市 B 嘉峪关市 C 酒泉市 D 张掖市 E 金昌市 F 武威市 G 白银市 H 定西市 I 天水市 J 平凉市 K 庆阳市 L 陇南市 M 临夏回族自治州 N 甘南藏族自治州

1. 您是否参与过如医学项目、试验等的医学科学研究？

A 是 B 否

1. 您是否接受过如健康讲座、调查等的健康科普教育？

A 是 B 否

1. 您是否在持续关注全球新型冠状病毒肺炎疫情的相关信息？

A 是 B 否

1. 单选题（请在您认为正确的选项序号上打“✔”，如不知道，请选D）
2. 邻近地区报道一例新型冠状病毒肺炎疑似感染患者，您认为以下做法正确的是：

A该地区和这个疑似感染者都与我无关，不必理会

B 如果这位疑似感染患者是我认识的人或我有亲朋好友在该地区，就会关注疫情

C 不管是否与我相关，都应对疫情进展信息保持持续关注

D 不知道

1. 新型冠状病毒肺炎疫情期间，获取我国新型冠状病毒肺炎预防、治疗和康复等相关信息最可信的途径是：

A 来源于国家或政府官方机构公布的信息

B 来源于非国家或政府官方机构公布的信息

C 来源于老百姓之间传播的信息

D 不知道

1. 某一项科学研究结果表明，某药物可以有效预防新型冠状病毒肺炎，您认为下面说法正确的是？

A 这是科学研究证实的，我会购买这种药

B 有些药会对某病毒或细菌有预防作用，我认为研究结果可信

C 这仅仅是一项科学研究的成果，我认为需要多项研究证实

D 不知道

1. 处于疫情高发地区的老张认为自己因经常吃大蒜所以没有感染新型冠状病毒，您认为吃大蒜可以预防新型冠状病毒肺炎吗？

A 可以，老张经常吃大蒜且没有感染

B 不确定，这只是老张的个人经验，不足以让人确信

C 不可以，没有任何方法可以预防

D 不知道

1. 某权威专家宣称某种药物可预防新型冠状病毒感染，但没有提供任何依据，您相信该药物可预防新型冠状病毒感染吗？

A 相信，因为这是权威专家推荐的

B 不相信，因为该专家没有提供可靠的依据

C 不相信，因为没有药可以预防

D 不知道

1. 科学研究发现，一种全新的方法可用于治疗新型冠状病毒肺炎，您觉得该新方法是否一定比之前的方法更有效？

A 不是，因为新的治疗方法不一定比旧的好

B 是，因为新的治疗方法一定比旧的好

C 是，因为新方法是建立在旧方法的基础上

D 不知道

1. 科学研究证明某药可有效预防季节性流感，因新型冠状病毒肺炎和流感症状相似，小王认为该药也会有效预防新型冠状病毒肺炎，您认为小王这种想法是否正确？

A 不确定，但理论上可认为该药预防新型冠状病毒肺炎有效

B 正确，流感和新型冠状病毒肺炎都是由病毒引起的，这药肯定有效

C 不正确，该药对预防流感有效，但不能直接推论到治疗新型冠状病毒肺炎有效

D 不知道

新型冠状病毒肺炎期间，某知名明星代言的广告宣称某品牌洗手液可以达到99%的病毒清除率，您相信吗？

A 相信，明星代言的一定真实可信

B 不相信，因为广告不代表科学研究

C 不相信，因为没有洗手液可以达到99%的病毒清除率

D 不知道

1. 某科学研究表明，通过有氧运动疗法共治疗5名新型冠状病毒肺炎轻症患者，其中4名患者康复，因此小李认为该有氧运动疗法可有效治疗新型冠状病毒肺炎，您认为小李这种想法是否正确？

A 不正确，研究只有5名患者，人数太少，暂时无法下结论

B 不正确，因为还有1名患者未康复

C 正确，因为有氧运动治疗后大部分患者都康复

D 不知道

1. 科学研究发现某药物在20～30岁新型冠状病毒肺炎患者中治疗效果很好，那么您认为该药物是否适用于70岁以上的新型冠状病毒肺炎患者？

A 适用，因为都是新型冠状病毒肺炎患者

B 不适用，因为这个药物只对20~30岁的患者有效

C 不确定，年龄上存在差异，其效果可能不一样

D 不知道

1. 科学研究发现某药在治疗感染新型冠状病毒的猫、狗等与人接触较为频繁和密切的动物身上有明显作用。能否说明该药对新型冠状病毒肺炎患者同样有作用？

A 不能，因为猫、狗与人的差别很大

B 能，动物的科学研究结果也是可靠的

C 能，该药对猫、狗有用，对人肯定也有用

D 不知道

1. 小李认为如果已知一种药物对预防新型冠状病毒肺炎有好处，那么多吃一些会帮助我们更好地预防。您认为小李的说法正确吗？

A 不正确，该药吃得更多预防效果不一定更好

B 正确，既然有好处，就应该多吃，多多益善

C 正确，既然有好处，多吃也无害

D 不知道

1. 情景题（请您阅读，然后回答相关问题。均为单选题，请在您认为正确的选项序号上打“✔”，如不知道，请选D）

国家卫健委发布的《新冠病毒疫苗接种技术指南（第一版）》中指出，对于18岁以下人群，目前已有的疫苗尚未获得用于该人群的临床试验数据，暂不推荐18岁以下人群接种。对于育龄期女性，如果在接种后怀孕或在未知怀孕的情况下接种了疫苗，基于对上述疫苗安全性的理解，不推荐仅因接种新冠病毒疫苗而采取特别医学措施（如终止妊娠），建议做好孕期检查和随访。对于有备孕计划的女性，不必仅因接种新冠病毒疫苗而延迟怀孕计划。

1. 各个社区正在统计接种疫苗的人群信息，小马家里有一个2岁的儿子，小马是否应该让孩子接种疫苗？

A 应该，可以预防新冠病毒肺炎

B 应该，谁都可以接种疫苗

C 不应该，孩子年龄不符合

D 不知道

1. 小马在接种疫苗后的第三天得知怀孕，是否应该终止怀孕？

A 应该，疫苗对孕妇和孩子不好

B 不应该，应做好孕期检查和随访

C 不应该，疫苗对孕妇孩子没有任何影响

D 不知道

English Version:

**Health Information Literacy of Chinese Residents with COVID-19 Questionnaire (2021)**

Dear Madame or Sir:

We are staff of the Health Commission of Gansu Province. We are conducting a survey to investigate the health information literacy of Chinese residents, which is a scientific research project of the Health Commission of Gansu Province. The information that you provide will be an important source of data for this study. Please read the following carefully before you participate in the survey.

All the data you provide will be used for research purposes only and will never be transmitted to any other person or organization. Therefore, please provide complete and accurate information.

There is no right or wrong answer: just complete the questions according to your understanding. Please complete all information yourself, independent of anyone else.

This study has been approved by the Ethics Review Committee of the School of Public Health, Lanzhou University. Your participation is very important to our research. Thank you so much for your assistance and participation. If you have any questions, please feel free to contact us. (Contact person: Jianqiang Wen; Contact number: 09318649683)

Best regards,

Health Information Literacy of Chinese Residents with COVID-19 Questionnaire (2021) Project team

**Ⅰ. Demographic Information**

1. **Date of birth:**
2. **Gender:** male or female
3. **Ethnicity:**
4. Han B. Hui C. Tibetan D. Mongolian E. Kazak F. Others
5. **Your education level:**
6. illiterate or barely literate
7. primary school
8. Secondary school
9. High School/ Professional high school/Special Secondary School
10. College
11. Bachelor’s degree
12. Master’s degree
13. Doctorate degree
14. **Your career:**
15. Professional (e.g., teachers / doctors / lawyers, etc.)
16. Service workers (e.g., caterers/drivers/salesmen, etc.)
17. Freelancers (e.g., writers / artists / photographers / tour guides, etc.)
18. Workers (e.g., factory workers/ construction workers/ urban sanitation workers, etc.)
19. Company employee
20. Government institution employee/ civil servants/ government staff
21. Student
22. Works in the home
23. Others (e.g., famers)

**6. Your household registration place:**

A. Urban B. Rural

**7. Your current residence city:**

**8. Have you been involved in medical science research? (e.g., medical projects, trials, etc.)**

A. Yes B. No

**9. Have you obtained health science education? (e.g., health lectures, surveys, etc.)**

A. Yes B. No

**10. Are you paying regular attention to the information of the COVID-19 pandemic?**

A. Yes B. No

**Ⅱ Single-choice questions (please mark "✔" in the beside the option you think is correct, if you do not know, please choose “D”)**

1. **If a suspected case of COVID-19 infection is reported in a neighboring area, what do you think is the correct approach:**
2. Ignore the case if either the area or this suspected infected person is not relevant to me.
3. I would be concerned about the case if the suspected infected person is someone I know or if I have friends or relatives in this area.
4. Regardless whether the suspected infected person is related to me or not, I would pay close attention to all information on the progress of the epidemic.
5. Don't know.
6. **The most credible way to obtain information related to the prevention, treatment, and rehabilitation of COVID-19 is:**
7. Information from official state or government agencies.
8. Information from non-state or official government agencies.
9. Information from the general public such as your relatives or neighbor.
10. Don't know.
11. **A scientific research study shows that a drug can effectively prevent COVID-19, what do you think is correct:**
12. Buy the drug and use it because it is scientifically proven.
13. Some drugs have a preventive effect against a virus or bacteria, and I think the study results are credible.
14. This is only the result of one scientific study and I think it needs to be confirmed by several studies before I would consider taking the drug.
15. Don't know.
16. **Zhang, who is in an area with a high prevalence of the epidemic, thinks he is not infected with the COVID-19 because he eats garlic regularly. Do you think eating garlic can prevent COVID-19?**
17. Yes, I think eating garlic can prevent COVID-19; Zhang will not get COVID-19.
18. I am not sure: this is only Zhang's personal experience; I don’t have enough information to be sure.
19. No, there is no way to prevent COVID-19.
20. Don't know.
21. **An authoritative expert claimed that a drug can prevent COVID-19, but did not provide any basis for this claim. Do you believe that the drug can prevent COVID-19?**
22. Yes, I believe the drug can prevent COVID-19 because it is recommended by an authoritative expert.
23. No, I don't believe the drug can prevent COVID-19 because the expert did not provide a reliable basis for his/her claim.
24. I don't believe this claim, because there are no effective medicines to prevent COVID-19.
25. Don't know.
26. **Scientific research has found a new approach to treating COVID-19. Do you think this new approach is necessarily more effective than currently available treatments?**
27. No, because the new treatment is not necessarily better than the old one.
28. Yes, because the new treatment is likely to be better than the old one.
29. Yes, because the new method is based on the old method to develop a new treatment.
30. Don't know.
31. **Scientific research has proven that a certain drug can effectively prevent seasonal influenza. Since the symptoms of COVID-19 and influenza are similar, Wang thinks that the drug will also effectively prevent COVID-19. Do you think Wang is right?**
32. I am uncertain, but theoretically the influenza drug should be effective in preventing COVID-19.
33. Yes, I think Wang is correct, as both influenza and COVID-19 are caused by viruses, therefore this drug should be effective against both diseases.
34. No, I think Wang is incorrect, the drug is effective in preventing influenza, but it cannot be directly inferred to be effective for treating COVID-19.
35. Don't know.
36. **During the COVID-19 pandemic, a celebrity-endorsed advertisement claimed that a brand of hand sanitizer could achieve kill 99% of the virus that causes COVID-19. Do you believe this claim?**
37. Yes, I believe, the celebrity endorsement must be true and credible.
38. No, I don't believe it: advertisements do not represent scientific research.
39. No, I don't believe it because no hand sanitizer can achieve kill 99% of the virus that causes COVID-19.
40. Don't know.
41. **One scientific research study showed that a total of five patients with mild cases of COVID-19 were treated with aerobic exercise therapy and four of them recovered. Therefore, Li believes that this aerobic exercise therapy can effectively treat COVID-19. Do you think Li is correct?**
42. No, I believe that Li is incorrect: there are only five patients in the study which is too small a study to draw conclusions from.
43. No, I believe that Li is incorrect, because the fifth patient did not recover.
44. Yes, I believe that Li is correct because most patients recovered after treatment with aerobic exercise.
45. Don't know.
46. **Scientific studies have found that a drug is effective in treating patients aged 20 to 30 years with COVID-19; do you think this drug is suitable for patients aged 70 years or older with COVID-19?**
47. Yes, the study is applicable to older patients because all are patients with COVID-19.
48. No, the study is not applicable because this drug is only effective in patients aged 20 to 30 years.
49. I am uncertain: there are differences in age and its effect may not be the same in the two groups.
50. Don't know.
51. **Scientific research has found that a drug has a significant beneficial effect on cats, dogs and other animals infected with COVID-19, who are in frequent and close contact with humans. Do you think that the drug has the same effect on patients with COVID-19?**
52. No, because cats and dogs are very different from people.
53. Yes, because the results of scientific studies on animals are also reliable.
54. Yes, because the drug benefits cats and dogs, it must work for humans too.
55. Don't know.
56. **Li thinks that if a drug is known to be effective for preventing COVID-19, then taking more of it will be even better. Do you think Li's statement is correct?**
57. I think Li is incorrect: the drug is not necessarily more effective if you take more of it.
58. I think Li is correct: since the drug has benefits, you should take more, the more the better.
59. I think Li is correct: since the drug has benefits, it is not harmful to take more.
60. Don't know.

**Ⅲ Scenario questions**

**Please read and then answer the subsequent questions. All are single-choice questions, please put "✔"beside the response option you think is correct, if you don't know, please choose option “D”.**

The Technical Guideline for Vaccination against COVID-19 (First Edition) issued by the National Health Commission states that for people under 18 years of age, clinical trial data for use in this population are not yet available for the currently available vaccines. Thus, vaccination is currently not recommended for people under 18 years of age. For women of childbearing age, if they become pregnant after vaccination or if they receive the vaccine in the case of unknown pregnancy, special medical measures (e.g., termination of pregnancy) are not recommended solely because of the COVID-19 vaccine based on the current understanding of the safety of the vaccine. For women who are planning to become pregnant, there is no need to delay pregnancy plans solely because of recent coronavirus vaccination.

1. **Each community is counting the vaccination population information. Ma has a 2-year-old son at home. Should the Ma vaccinate the child?**
2. Yes, because vaccination can prevent COVID-19.
3. Yes, because anyone can be vaccinated.
4. No, the child should not be vaccinated because vaccination is not currently recommended in his age group.
5. Don't know
6. **Ma is informed of pregnancy on the third day after vaccination. Should the pregnancy be terminated?**
7. Yes, I believe that pregnancy should be terminated: vaccines are not good for pregnant women and children.
8. No, pregnancy should not be terminated: careful pre-natal care and follow-up should be performed.
9. No, pregnancy should not be terminated: the vaccine does not affect pregnant women and their unborn children.
10. Don't know
